# Insulin resistance and associated factors in female adolescents from two capital cities in the north and south of Brazil

**DOI:** 10.1186/s13098-021-00730-8

**Published:** 2021-10-19

**Authors:** Ivanice Fernandes Barcellos Gemelli, Thais Rasia Silva, Edson dos Santos Farias, Maria Teresa Anselmo Olinto, Poli Mara Spritzer

**Affiliations:** 1 Federal University of Rio Grande Do Sul (UFRGS), Porto Alegre, Brazil; 2 Federal University of Rondônia, Rondônia, Brazil; 3 Gynecological Endocrinology Unit, Division of Endocrinology, Hospital de Clínicas de Porto Alegre (HCPA), Porto Alegre, Brazil; 4 University of Vale Do Rio Dos Sinos, UNISINOS, São Leopoldo, Brazil; 5 Department of Physiology, Laboratory of Molecular Endocrinology, Federal University of Rio Grande Do Sul (UFRGS), Porto Alegre, Brazil; 6 Division of Endocrinology, Hospital de Clínicas de Porto Alegre, Rua Ramiro Barcelos 2350, Porto Alegre, Rio Grande Do Sul 90035-003 Brazil

**Keywords:** Menarche, Insulin resistance, Puberty, Adolescent, Obesity

## Abstract

**Background:**

It has been described that physiological changes in glucose metabolism, represented by insulin resistance (IR), are predicted during pubertal evolution, and obesity may be associated with its persistence even at the end of puberty. The aim of this study was to investigate the prevalence of IR in female adolescents with possible associated factors and evaluate the relationship of time since menarche (< 2 vs. ≥ 2 years) in the occurrence of IR in two Brazilian capital cities: Porto Velho (RO) and Porto Alegre (RS).

**Methods:**

This is a cross-sectional school-based study, using information from the Study of Cardiovascular Risks (ERICA) database for adolescents aged 12–17 years, enrolled in public and private schools, in municipalities with more than 100,000 inhabitants in Brazil, between 2013 and 2014. The present study included 889 adolescents, 382 in Porto Velho (PVh) and 507 in Porto Alegre (PoA). The homeostasis model assessment for insulin resistance (HOMA-IR) ≥ 3.16 and fasting insulin ≥ 15 mU/L was used to determine the outcome variable of IR. Estimates of crude and adjusted prevalence ratios with confidence intervals of 95% were calculated using Poisson regression with robust variance. Sociodemographic, behavioral, reproductive and nutritional characteristics were considered as potential confounding factors in multivariable models based on a conceptual framework of IR determination.

**Results:**

In the total sample, the prevalence of IR was 22.03% (95% CI 17.84–26.89). After adjusting the models, age 15–17 years and time since menarche ≥ 2 years were found to act as protective factors for IR; in contrast, the highest probability of IR was observed in black adolescents, with increased waist circumference (WC) and overweight/obesity (Ow/Ob). The protective effect of two or more years since menarche (post-menarche) was observed for both higher HOMA-IR and fasting insulin in PVh; in PoA, such protection was maintained only for fasting insulin ≥ 15 mU/L after adjustments in the multivariate models.

**Conclusions:**

IR is more prevalent during the peri-menarche period, especially in younger and black adolescents, compared to their white and post-menarche counterparts. The association between Ow/Ob and high WC with the occurrence of IR was independent of age and ethnicity variables.

**Supplementary Information:**

The online version contains supplementary material available at 10.1186/s13098-021-00730-8.

## Background

Insulin resistance (IR) is well reported to occur during puberty but the underlying mechanisms of physiological changes in glucose metabolism at this period are not entirely established [1–5]. Longitudinal studies have reported a transitory IR during pubertal evolution and a trend toward recover at the end of puberty [1–6].

It has also been shown that obesity not only affects the reduction in insulin sensitivity during puberty from early stages, but may also be associated with its persistence in the years following puberty [5, 6]. Given the global high prevalence of overweight and obesity in childhood and adolescence [7], this is a clinically relevant issue, since obesity and IR in young individuals are risk factors for pre-diabetes and diabetes mellitus (DM) in adulthood [2–4, 8].

While the relationship between puberty and IR has been reported in different studies, IR and time of menarche have been addressed less frequently [9], although emerging evidence suggests an association between excess weight and age at menarche [10, 11]. According to studies conducted in Brazil, it differs from other countries in being such a large country, with significant demographic, cultural and ethnic diversity among its regions, such as in the north and south of the country [12, 13].

In face of the current worldwide prevalence of obesity, pubertal changes in glucose metabolism, and the fact that adolescents in northern Brazil are part of a less-represented studied group, which in contrast to the south is less urbanized, the aim of this study was to investigate the prevalence of IR in female adolescents and to evaluate the relationship with time since menarche (< 80.2 vs. ≥ 2 years) and possible associated factors in the occurrence of IR.

## Materials and methods

### Study design and participants

This is a cross-sectional study that used data from two centers, part of the Study of Cardiovascular Risks in Adolescents (ERICA 2013–2014). ERICA is a school-based, national, multicenter and cross-sectional study carried out in rural and urban contexts. The design of the ERICA study has been published previously (2015) [14]. Briefly, 73,624 students aged 12–17 years were enrolled from private and public schools, located in one of the 273 Brazilian municipalities with more than 100,000 inhabitants [15]. For the present study, all female students from Porto Velho-RO (PVh) and Porto Alegre-RS (PoA), who participated in all research stages of ERICA and had already had menarche, were included.

This study was approved by the Research Ethics Committee (REC) of the Federal University of Rondônia, Federal University of Rio Grande do Sul and the Institute of Studies in Collective Health of the University of Rio de Janeiro (Protocol 45/2008), and was conducted according to the principles of the Helsinki declaration [16]. Written informed consent was obtained from each student and from his or her parents. The present study included a subsample of the students residing in two capitals: Porto Velho (PVh) and Porto Alegre (PoA), respectively, located in the Southern and Northern regions of Brazil.

### Data collection

A self-administered questionnaire using a personal digital assistant (PDA, model LG GM750Q) was administered. Data regarding sociodemographic, behavioral and diet characteristics were obtained. The economic status was defined according to the Brazilian Association of Companies and Research (ABEP in the Portuguese acronym), as A1 (the highest social class), A2, B1, B2, C1, C2, D and E (the lowest social class) [17] data were grouped into 3 categories: A, B and C/D. Age was collected in full years and further grouped (12–13, 14, and 15–17 years. Ethnicity was defined by skin color as white and non-white (black, mixed or indigenous) [18, 19]. Smoking and alcohol consumption were assessed according to whether participants had already experimented them or not [19–21]. Physical activity was categorized as inactive (students with no leisure-time, physical activity, or who exercised less than 300 min/week), or active for those who exercised from ≥ 300 to 1200 min/week) [22]. Recommended screen time was up to 2 h per day, and not recommended was more than 2 h per day, according to the American Academy of Pediatrics guidelines [23]. Menarche was assessed according to age (age at menarche 9–12, or 13–16 years) and time since menarche, that is, peri-menarche: less than 2 years since menarche, and post-menarche: 2 years or more since menarche. This classification considered the expected time for maturation of the reproductive axis, after menarche, accepted as being more than 2 years of occurrence of the menarche event [24].

As for nutritional status, body mass index (BMI) was used. Height was assessed using a portable and demountable stadiometer, Alturexata^®^ [25]. Body weight was assessed using a digital scale from Leader, model P150m, capacity of 200 kg and precision of 50 g. BMI was defined by weight (kilograms) divided by square of the height (meters). The girls were stratified by overweight and obesity (z-score > 1) and normal weight groups (z-score ≤ 1), according to BMI-for-age z-scores from the World Health Organization child growth standards [26]. Waist circumference was measured to the nearest 1 mm using a fiber glass anthropometric tape, with millimeter resolution and length of 1.5 m (Sanny^®^, São Paulo, Brazil). WC classification followed the International Diabetes Federation (IDF) guidelines, which uses the 90th percentile as a cutoff point for girls up to 16 years old and 80 cm for those over 16 years old [27].

Dietary intake was assessed using a 24-h recall performed by trained interviewers. The food and drinks consumed were recorded in all meals and snacks before the interview in the dietary assessment software, ERICA-REC 24 h [28]. Portion size estimation was obtained by showing photographs included in the software. Nutritional composition was calculated using the software database consisted of 1626 food items based on data from a Dietary National Survey carried out from 2008 to 2009 [29]. Energy and nutrients were estimated using the IBGE table [30].

Specifically for this study, nutritional characterization followed the dietary reference intake (DRI) [31] and presented total energy intake (Kcal), percentage of trans fatty acid (TFA), and ratio of omega-6 to omega-3 fatty acids. The three components were characterized according to mean intake in PVh and PoA.

### Outcome assessments

Blood samples were collected after 12 h overnight fasting. Glucose was measured by the hexoquinase method; triglycerides, by enzymatic kinetics and HDL-cholesterol by enzymatic colorimetric assay (ADVIA 2400, Siemens). LDL- cholesterol was calculated by the Friedewald equation. Insulin was determined by chemiluminescence method (Modular Analytics-Roche) [32]. IR was calculated using the model of insulin homeostasis, HOMA-IR Index as follows: insulin (mU/L) × (glucose (mg/dL) × 0.0555)/22.5, as proposed by Matthews et al. [33]. The ≥ 3.16 cutoff point, according to the first guidelines for the prevention of atherosclerosis in childhood and adolescence [31], was used in our analysis. Fasting insulin, with a cutoff of ≥ 15 mU/mL was also assessed as an additional marker of IR [34].

### Statistical analysis

Demographic, nutritional, anthropometric, and biochemical variables were expressed as a percentage and 95% confidence interval (CI). The differences between the cities were assessed using the student *t* test for continuous variables and the chi-square test for dichotomous variables.

**Fig. 1:**
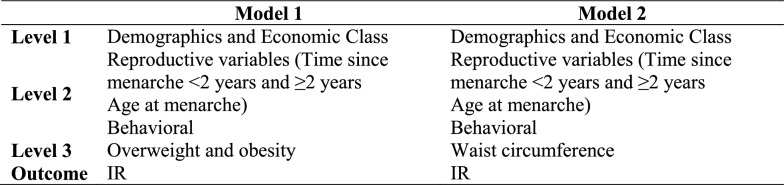
Conceptual model

All factors with IR associated were converted to categorical variables to enable the comparability of prevalence ratios (PRs). In the evaluation of unadjusted and adjusted measures of effect in the multivariate models, Poisson regression with robust variance was used. The adjusted analysis followed a conceptual model defined a priori [35]. Variables that were associated with outcomes at a significance level of ≤ 20% in the unadjusted analysis were included in the multivariate model as potential confounders. At level I, the most distal level of determination, sociodemographic variables were included; at level II, the reproductive and behavioral ones; and at level III, the most proximal, the nutritional status variables. Finally, variables with a p value of ≤ 0.05 were considered associated with the outcomes, that is, IR (insulin levels and HOMA-IR). Due to the collinearity between WC and overweight/obesity (Ow/Ob), these two variables of nutritional status were entered into different models, model 1 and model 2, respectively (shown in Fig. 1).

In addition, the multivariate-adjusted Poisson regression model with robust estimates was used to assess the association among the subgroups of time since menarche (peri-menarche and post-menarche) in each city with demographic, lifestyle, anthropometric factors and IR.

Statistical analyzes were performed using the statistical software STATA, version 14 (Stata Corporation, College Station, TX, USA).

## Results

A total of 889 female adolescents between 12 and 17 years of age were included, 382 in PVh and 507 in PoA, mean age was 14.27 (95% CI 14.21–14.34) and 14.58 years (95% CI 14.50–14.65) in the north and south of Brazil, respectively. Table 1 shows a comparison of the sample between the two capitals, according to sociodemographic, reproductive, behavioral, nutritional status and food consumption variables. There was a higher percentage of older adolescents (15–17 years), of adolescents classified as white, and belonging to economic class A in the city of PoA, when compared to PVh. Although, in both cities, almost 60% of adolescents belonged to economic class B. Regarding behavior, adolescents in PoA presented a higher prevalence of smoking, alcohol consumption and screen time compared to those in PVh.

**Table 1:** Characterization of adolescents in the sample by capital, according to sociodemographic, reproductive, behavioral and nutritional variables, Porto Velho and Porto Alegre, 2013/2014 (n = 889)

Characteristics (889)	n	Porto Velho (382)	n	Porto Alegre (507)	p-value
		% (95% CI)		% (95% CI)	
Sociodemographic characteristics					
Chronological age					
12–13 years	94	36.51 (34.62–38.45)	97	30.22 (28.24–32.27)	<** 0.001**
14 years	74	19.83 (18.68–21.02)	117	17.75 (17.12–18.39)	
15–17 years	214	43.66 (42.19–45.14)	293	52.03 (50.47- 53.59)	
Ethnicity					
White	120	27.77 (23.14–32.92)	359	67.27 (58.5–74.97)	**< 0.001**
Black	13	3.14 (1.8–5.41)	38	11.54 (7.31–17.74)	
Other	238	69.09 (63.27–74.37)	104	21.2 (15.88–27.7)	
Economic class					
A	22	6.51 (3.4–11.79)	76	17.36 (9.26–30.17)	**0.019**
B	171	57.83 (47.84–67.22)	227	59.12 (50.54–67.18)	
C/D	93	35.66 (25.66–47.09)	89	23.52 (17.81–30.4)	
Reproductive characteristics					
Age at menarche					
9–12 years	278	76.42 (71.88–80.43)	382	79.95 (75.44–83.81)	0.241
13–16 years	104	23.58 (19.57–28.12)	125	20.05 (16.19–24.56)	
Time of menarche					
< 2 years	91	32.34 (28.36–36.59)	91	25.47 (20.01–31.83)	0.069
≥ 2 years	291	67.66 (63.41–71.64)	416	74.53 (68.17–79.99)	
Behavioral characteristics					
Smoking					
Yes	71	17.4 (13.6–22)	147	30.08 (23.83–37.17)	**0.001**
No	311	82.6 (78–86.4)	360	69.92 (62.83–76.17)	
Alcohol					
Yes	181	47.01 (42.06–52.02)	381	79.51 (72.78–84.92)	**0.001**
No	173	52.99 (47.98–57.94)	97	20.49 (15.08–27.22)	
Screen hours					
< 2 h/day	177	54.5 (47.56–61.27)	133	31.21 (25.56–37.49)	**< 0.001**
≥ 2 h/day	172	45.5 (38.73- 52.44)	327	68.79 (62.51–74.44)	
Physical activity					
Active	156	44.16 (36.44–52.17)	186	34.34 (28.48–40.73)	*0.053*
Inactive	205	55.84 (47.83–63.56)	301	65.66 (59.27–71.52)	
Nutritional status					
Nutritional status (%)					
Low weight and normal weight	294	77.11 (69.17–83.49)	345	65.68 (58.59–72.13)	**0.028**
Overweight/obesity	88	22. 89 (16.51–30.83)	162	34.32 (27.87–41.41)	
Waist circumference					
Normal	332	87.93 (81.66–92.26)	423	84.39 (80.17–87.85)	*0.296*
Elevated	50	12.07 (7.74–18.34)	84	15.61 (12.15–19.83)	

Diet		Mean (standard error)		Mean (standard error)	
Energy (Kcal)		2238.617 (85.43)		1923.761 (60.83)	**0.005**
Omega-6: omega-3 ratio		8.21 (0.20)		8.97 (0.40)	*0.099*
Trans-fat		0.91 (0.77)		1.26 (0.63)	**0.001**

Student *t* test, mean and standard error and chi-square, 95%CI. Smoking: yes = already experimented; Alcohol: yes = already experimented; Physical activity: inactive = 0–300 min/wk.; Screen hours: not recommended = more than 2 h/day; Nutritional status classification according to BMI-for-age z-score (WHO, [49]); Waist circumference; Altered blood glucose (IDF, [48]); Energy expressed by daily intake in Kcal, Omega-6: Omega-3 ratio and Trans-fat cutoff point established by the dietary reference intakes (DRI, [31]). Eco Class D: Corresponded to 1.04% of the total sample (2.21% of the sample in Porto Velho and 0.67% in Porto Alegre) Sample N: Ethnicity n = 872, economic class n = 678, alcohol n = 832, physical activity n = 848

Statistically significant differences between capitals are in bold (*p* < 0.05)

Also in Table 1, it is observed that Ow/Ob was also higher in PoA compared to PVh. As for the indicators used for quality of diet, there was a higher caloric intake in PVh, but a higher mean consumption of trans-fat in PoA.

The prevalence of HOMA-IR (≥ 3.16) in the total sample was 22.03% (95% CI 17.84–26.89), being 18.22% (95% CI 11.69–27, 27) and 23.33% (95% CI 18.32–29.21), in PVh and PoA, respectively—data not shown in the table.

Table 2 shows the prevalence and PRs for IR (HOMA-IR ≥ 3.16), according to the characteristics of the sample. In both capitals, there is a protective relationship for IR in the group of adolescents aged 15–17 years, compared with the younger ones, as well as in the group with time since menarche of two or more years, compared with those with time since menarche of less than two years, being 54% and 45% in PVh and PoA, respectively. This protection corresponded to 47% when considering the total sample (Additional file 1: Table S1). Also in both capitals, girls with Ow/Ob and those with altered WC were more likely to have IR. Difference in relation to ethnicity was observed only in the southern capital, PoA, where IR was more likely to occur in black adolescents.

**Table 2:** Prevalence and prevalence ratio of insulin resistance according to sociodemographic, reproductive, behavioral and nutritional characteristics, Porto Velho and Porto Alegre, 2013/2014 (n = 889)

Characteristics	HOMA-IR ≥ 3.16					
	Porto Velho			Porto Alegre		
	% (CI)	PR (CI)	PR p value	% (CI)	PR (CI)	PR p value
Sociodemographic characteristics						
Chronological age						
12–13 years	25.26 (13.64–41.97)	1	1	32.2 (21.98–44.47)	1	1
14 years	16.7 (7.66–32.65)	0.66 (0.31–1.41)	0.263	21.12 (12.65–33.11)	0.65 (0.36–1.16)	0.144
15–17 years	12.95 (7.87–20.58)	**0.51 (0.28–0.92)**	**0.028**	18.84 (12.96–26.57)	**0.58 (0.35–0.95)**	**0.035**
Ethnicity						
White	18.34 (9.89–31.48)	1	1	17.48 (11.89–24.95)	1	1
Black	14.54 (1.98–58.84)	0.80 (0.11–5.71)	0.818	45.49 (27.33–64.94)	**2.59 (1.36–4.94)**	**0.005**
Other	18.97 (11.54–29.59)	1.03 (0.59–1.77)	0.906	29.77 (21.57–39.52)	**1.69 (1.03–2.79)**	**0.038**
Economic class						
A	14.96 (4.59–39.15)	1	1	16.55 (6.44–36.33)	1	1
B	19.64 (13.71–27.33)	1.22 (0.26–5.55)	0.781	27.19 (21.11–34.26)	1.63 (0.55–4.83)	0.357
C/D	17.22 (7.22–35.75)	1.05 (0.17–6.57)	0.949	23.49 (13.13–38.42)	1.42 (0.38–5.19)	0.580
Reproductive characteristics						
Age at menarche						
9–12 years	19.91 (12.26–30.65)	1.59 (0.75–3.36)	0.207	23.39 (17.94–29.9)	1.02 (0.62–1.67)	0.921
13–16 years	12.54 (5.61–25.68)	1	1	22.84 (13.94–35.1)	1	1
Time of menarche						
< 2 years	28.31 (13.43–50.14)	1	1	34.96 (25.11–46.29)	1	1
≥ 2 years	13.34 (8.73–19.85)	**0.46 (0.24–0.90)**	**0.027**	19.28 (13.99–25.98)	**0.55 (0.35–0.84)**	**0.008**
Behavioral characteristics						
Smoking						
Yes	29.76 (13.69–53.09)	1.89 (0.79–4.48)	0.136	20.1 (13.67–28.67)	0.81 (0.48–1.35)	0.414
No	15.76 (8.99–26.16)	1	1	24.64 (17.69–33.23)	1	1
Alcohol						
Yes	23.11 (14.61–34.54)	1.48 (0.90–2.43)	0.110	21.76 (16.09–28.75)	0.67 (0.42–1.07)	0.097
No	15.47 (8.96–25.4)	1	1	31.89 (20.68–45.68)	1	1
Screen hours						
< 2 h/day	24.46 (15.14–36.76)	1	1	19.48 (11.87–30.31)	1	1
≥ 2 h/day	18.26 (12.67–25.6)	0.75 (0.43–1.32)	0.313	25.38 (18.9–33.17)	1.29 (0.71–2.37)	0.378
Physical activity						
Active	18.5 (11.48–28.42)	1	1	21.47 (15.48–29)	1	1
Inactive	17.55 (9.81–29.39)	1.05 (0.56–1.93)	0.865	22.02 (15.21–30.78)	0.97 (0.61–1.56)	0.928
Nutritional status						
Nutritional status (%)						
Low weight and normal weight	9.48 (4.51–18.84)	1	1	18.84 (13.76–25.26)	1	1
Overweight/obesity	48 (37.28–58.82)	**5.04 (2.53–10.05**)	**< 0.001**	31.77 (24.81–39.66)	**1.68 (1.26–2.24)**	**0.001**
Waist circumference						
Normal	12.47 (6.87–21.56)	1	1	20.05 (15.22–25.94)	1	1
Elevated	61.21 (42.81–76.89)	**4.90 (2.53–9.48)**	**< 0.001**	40.69 (27.41–55.48)	**2.02 (1.37–2.99)**	**0.001**

Chi-square test 95% CI. Robust Poisson regression p value < 0.05; Time since menarche < 2 years since the occurrence of menarche, and ≥ 2 years since the occurrence of menarche; Ethnicity: white, black and other (indigenous, mixed and yellow). Smoking: yes = already experimented; Alcohol: yes = already experimented; Physical activity: inactive = 0 to 300 min/wk.; Screen hours: not recommended = more than 2 h/day Nutritional status classification according to BMI-for-age z-score (WHO, [49]); Waist circumference (IDF, [48]); HOMA-IR ≥ 3.16 (I Diretriz de prevenção da aterosclerose na infância e adolescência, [50])

Statistically significant prevalence ratios are in bold (*p* < 0.05)

The prevalence of hyperinsulinemia (insulin ≥ 15 mU/mL) in the total sample was 6.52% (95% CI 4.35–9.65), being 8.41% (95% CI 4.54–15.09) and 5.87% (95% CI 3.49–9.72), in PVh and PoA, respectively—data not shown in the table.

Table 3 shows the prevalence and PRs for hyperinsulinemia, according to the characteristics of the sample. There is a protective relationship for the group of adolescents with time since menarche of two or more years, compared to those with less than two years since menarche, observed in PoA. This relationship was of marginal statistical significance in PVh. Hyperinsulinemia were also more likely to occur in adolescents classified as black in PoA. Adolescents with behavioral characteristics, such as experimenting with tobacco and alcohol, were more likely to present hyperinsulinemia only in PVh.

**Table 3:** Prevalence and prevalence ratio of hyperinsulinemia according to sociodemographic, reproductive, behavioral and nutritional characteristics, Porto Velho and Porto Alegre, 2013/2014 (n = 889)

Characteristics	Insulin ≥ 15 mU/mL					
	Porto Velho			Porto Alegre		
	% (CI)	PR (CI)	PR p value	% (CI)	PR (CI)	PR p value
Sociodemographic characteristics						
Chronological age						
12–13 years	21.1(11.05–6.53)	1		27.3 (16.69–41.32)	*1*	
14 years	17.77(6.61–39.74)	0.84 (0.32–2.21)	0.711	19.42 (11.78–30.31)	0.71 (0.34–1.44)	0.334
15–17 years	13.41(8.33–20.91)	0.63 (0.34–1.17)	0.135	15.86 (11.5–21.48)	0.58 (0.32–1.03)	0.062
Ethnicity						
White	16.13(8.25–29.14)	1		15.25 (10.55–21.54)	1	
Black	14.55(1.99–58.84)	0.91 (0.13–6.34)	0.926	40.06 (21.14–64.04)	**2.68 (1.23–5.81)**	**0.015**
Other	18.21(10.77–29.11)	1.12 (0.58–2.17)	0.708	23.53 (15.58–33.9)	1.53 (0.85–2.76)	0.147
Economic class						
A	12.05(4.19–30.01)	1		14.91 (5.55–34.34)	1	
B	19.75(13.65–27.71)	1.55 (0.35–6.72)	0.532	25.65 (19.68–32.7)	1.71(0.51–5.65)	0.361
C/D	18.86(8.06–38.14)	1.45 (0.25–8.18)	0.652	19.78 (11.72–31.4)	1.32 (0.35–4.92)	0.661
Reproductive characteristics						
Age at menarche						
9–12 years	18.91(11.57–29.37)	1.70 (0.79–3.69)	0.160	20.7 (15.45–27.17)	1.21 (0.68–2.16)	0.484
13–16 years	11.05(4.7–23.82)	1		16.96 (10.56–26.1)	1	
Time of menarche						
< 2 years	23.92(1.18–42.48)	1		32.7 (22.62–44.68)	1	
≥ 2 years	13.82(8.79–21.08)	0.57 (0.32–1.02)	0.061	15.59 (11.72–25.08)	**0.47 (0.30–0.74)**	**0.002**
Behavioral characteristics						
Smoking						
Yes	31.78(15.27–54.64)	**2.27 (1.09–4.73)**	**0.030**	16.1 (10.11–24.65)	0.74 (0.40–1.36)	0.327
No	14(8.2–26.42)	1		21.61 (15.39–29.47)	1	
Alcohol						
Yes	22.04(13.51–3.85)	**1.52 (1.04–2.23)**	**0.031**	20.19 (15.05–26.54)	0.79 (0.49–1.29)	0.344
No	14.29(8.71–22.58)	1		25.16 (15.86–37.49)	1	
Screen hours						
< 2 h/day	21.94(14.02–2.63)	1		17.84 (10.4–28.88)	1	
≥ 2 h/day	18.04(11.11–7.94)	0.83 (0.46–1.50)	0.537	22.34 (16.62–29.33)	1.23 (0.63–2.40)	0.513
Physical activity						
Active	17.66(11.41–6.31)	1		21.33(15.12–29.21)	1	
Inactive	15.94(8.2–28.7)	1.10 (0.56–2.17)	0.747	16.76 (11.59–23.61)	1.27 (0.78–2.07)	0.308
Nutritional status						
Nutritional status (%)						
Low weight and normal weight	8.2(3.72–17.13)	1		14.76 (10.54–20.29)	1	
Overweight/obesity	47.17(35.27–9.39)	**5.74 (2.68–12.28**)	**< 0.001**	29.92 (22.59–38.45)	**2.02 (1.39–2.93)**	**0.001**
Waist circumference						
Normal	11.55(6.45–19.83)	1		16.39 (11.74–22.4)	1	
Elevated	57.91(36.52–76.7)	**5.00 (2.54–9.8)**	**< 0.001**	39.19 (26.25–53.85)	**2.38 (1.42–4.01)**	**0.002**

Chi-square test 95%CI. Robust Poisson regression p value < 0.05; Time since menarche < 2 years since the occurrence of menarche, and ≥ 2 years since the occurrence of menarche; Ethnicity: white, black and other (indigenous, mixed and yellow). Smoking: yes = already experimented; Alcohol: yes = already experimented; Physical activity: inactive = 0–300 min/wk.; Screen hours: not recommended = more than 2 h/day Nutritional status classification according to BMI-for-age z-score (WHO, [49]); Waist circumference (IDF, [48]); Insulin ≥ 15 mU/mL (I Diretriz de prevenção da aterosclerose na infância e adolescência, [50])

Statistically significant prevalence ratios are in bold (*p* < 0.05)

As for nutritional status, Ow/Ob and greater WC showed an increased probability for the occurrence of hyperinsulinemia in the two capitals. The strength of the association was greater in adolescents in PVh for the two outcomes, IR and hyperinsulinemia (Tables 2 and 3).

Table 4 shows the PR for IR, after adjustment in the multivariate models in both capitals. The age of 15–17 years, that is, late adolescence and the time since menarche ≥ 2 years were protective factors for both HOMA and hyperinsulinemia; on the other hand, the highest probability of both outcomes was observed both in black adolescents, with greater WC and Ow/Ob. City-specific multivariate models are available in Additional file 1: Tables S2 and S3.

**Table 4:** Adjusted prevalence ratio of factors associated with insulin resistance in the total sample

	Porto Velho and Porto Alegre (889)			
	HOMA-IR		Insulin	
	Adjusted	P value	Adjusted	P value
Level 1				
Age				
12–13 years	1		1	
14 years	0.72 (0.45–1.15)	0.173	0.82 (0.45–1.48)	0.508
15–17 years	0.59 (0.40–0.88)	**0.012**	0.62 (0.40–0.96)	**0.036**
Ethnicity				
Black	2.36 (1.29–4.31)	**0.006**	2.47 (1.20–5.08)	**0.015**
Others	1.28 (0.85–1.91)	0.217	1.27 (0.81–1.99)	0.289
Level 2				
Time since menarche				
≥ 2 years	0.64 (0.42–0.97)	**0.039**	0.53 (0.37–0.76)	**0.001**
Level 3				
Model 1				
Waist circumference				
Altered	2.58 (1.97–3.38)	**< 0.001**	2.95 (2.08–4.19)	**< 0.001**
Model 2				
Nutritional status				
Overweight and obesity	2.08 (1.58–2.73)	**< 0.001**	2.50 (1.81–3.46)	**< 0.001**

Poisson regression; P value ≤ 0.05 and 95% CI; IR = HOMA ≥ 3.16, Insulin ≥ 15 mU/mL (I Diretriz de prevenção da aterosclerose na infância e adolescência, [50]). Model 1 included waist circumference and model 2 included overweight and obesity; Ethnicity: white (reference), black and others (indigenous, mixed and yellow); Group Menarche ≥ 2 years: 2 years or more since menarche; Reference: category = 1 (Less than 2 years since menarche); altered waist circumference > 90th percentile or ≥ 80 cm (IDF, [48]); nutritional status classification according to BMI-for-age z-score (WHO, [49])

Statistically significant prevalence ratios are in bold (*p* < 0.05)

Table 5 presents adjusted models, stratified by PVh and by PoA comparing time since menarche. The protective effect of 2 years or more since menarche was observed for both HOMA-IR and fasting insulin in PVh, even after adjustments in the four models. In PoA only in the unadjusted model, protection for HOMA-IR was present, however, for fasting insulin such protection was maintained even after adjustment for age, ethnicity, smoking, alcohol, WC and Ow/Ob.

**Table 5:** Prevalence ratios of insulin resistance, altered HOMA-IR and hyperinsulinemia, according to time since menarche

	Porto Velho (382)	p value	Porto Alegre (507)		p value
	Post-menarche		Peri-menarche	Post-menarche	
	PR		Reference	PR	
HOMA-IR					
Mod. 1	0.46 (0.21–1.04)	0.062	1	0.55 (0.34–0.87)	**0.013**
Mod. 2	0.60 (0.31–1.16)	0.122	1	0.64 (0.34–1.20)	0.160
Mod. 3	0.53 (0.29–0.96)	**0.038**	1	0.64 (0.38–1.07)	0.090
Mod. 4	0.49 (0.24–1.00)	**0.050**	1	0.64 (0.78–1.05)	0.079
Insulin					
Mod. 1	0.57 (0.27–1.18)	**0.125**	1	0.47 (0.29–0.77)	**0.005**
Mod. 2	0.68 (0.35–1.30)	0.230	1	0.54 (0.31–0.93)	**0.031**
Mod. 3	0.62 (0.40–0,94)	**0.030**	1	0.54 (0.32–0.88)	**0.017**
Mod. 4	0.56 (0.34–0.94)	**0.030**	1	0.53 (0.32–0.87)	**0.015**

Data are expressed as prevalence ratio and 95% confidence interval (CI); Poisson regression to Model 1; Group peri-menarche: Less than 2 years since menarche; Group post-menarche: 2 years or more since menarche; Multivariate Poisson regression to models adjusted for chronological age (increase of 1 year); ethnicity (non-white), smoking (experimented), alcohol (experimented), waist circumference and nutritional status (overweight and obesity); PR = Prevalence ratios (menarche B in relation to menarche A); p value ≤ 0.05 and 95% CI; Altered HOMA-IR: ≥ 3.16; hyperinsulinemia: > 15 mU/mL; Model 1—unadjusted; Model 2—adjusted for age and ethnicity; Model 3—adjusted for age, ethnicity, smoking, alcohol, and waist circumference; Model 4—adjusted for age, ethnicity, smoking, alcohol and nutritional status

Statistically significant prevalence ratios are in bold (*p* < 0.05)

Supplementary material also shows the distribution of dietary components according to the presence of IR in the two capitals (Additional file 1: Table S4), followed by prevalence of IR according to time since menarche (Additional file 2: Table S5).

## Discussion

In the present study with adolescents from two Brazilian regions, the prevalence of IR was 22% and age between 15 and 17 years and time since menarche ≥ 2 years were protective factors for IR. In turn, the highest probability of IR was observed in black adolescents, with increased WC circumference and Ow/Ob.

The prevalence of IR has been assessed in other countries, and a wide range of values (from 3.1% in Greece to 44% in New Zealand) has been reported. Indeed, specific characteristics in each study, such as the age of the adolescent population and criteria used to define IR at each age may have influenced on this variation [31]. Similar to our findings, one study with Brazilian female adolescents using the 75th percentile of the HOMA-IR found a 27.8% prevalence of IR [9].

In this sense, in the current study, the general sample involved both eutrophic and Ow/Ob adolescents, which may have impacted on the prevalence of IR [7] Besides, the prevalence of IR may have been further attenuated as a result of the choice of the HOMA cutoff point, as shown in a meta-analysis, in which the lowest prevalence was demonstrated in a study that used a HOMA cutoff of 3.16, and when using 2.1, the prevalence increased twofold [36].

Interestingly, we found no difference in the prevalence of IR among female adolescents living in the two Brazilian regions, with notorious environmental and cultural diversity. The similarity of the outcome between the two capitals is in line with other studies that described IR as physiological process during puberty [1–6].

Knowledge of factors associated with IR, as well as the identification of population groups at higher risk for chronic non-communicable diseases, is a matter of concern in adolescence [37]. Here, we present robust results of factors associated with IR showing that Ow/Ob and WC increased the probability of IR by around five times when compared to eutrophic teenagers.

In fact, changes in insulin sensitivity during puberty seem to be worsened by obesity [2] and the expectation of recovery in insulin sensitivity following puberty, may not occur in obese girls [1–3]. The non-occurrence of insulin sensitivity recovery is supported by the fact that the hypertrophic adipocytes are more susceptible to inflammation, apoptosis, fibrosis, and release of free fatty acids, which is associated with IR [2, 38]. Moreover, central obesity is closely related to glucose intolerance and IR and girls with high WC are at greater risk for type 2 diabetes and cardiometabolic comorbidities than girls with normal WC at the same age [1, 2]. Thus, our findings suggest that adolescents who did not maintain protection after menarche and who were obese, were exposed to these comorbidities early on.

Currently, studies involving puberty that provide data on IR, most often include Tanner staging or pre-pubertal and pubertal periods [37]. In the present study, we analyzed adolescents from the peri-menarche period, presenting higher prevalence of IR in both capitals, when compared with the group in the post-puberty, with 2 years or more since menarche, a period in which insulin sensitivity could be expected to return [39]. We also found that younger, black adolescents in PoA had a longer exposure to IR.

Unhealthy lifestyle habits are related to metabolic risks in childhood and adolescence [40–42]. Here, we report that in the southern capital, adolescents had less healthy habits, such as being more inactive, with more experimental use of tobacco and alcohol, in addition to having a higher intake of trans-fatty acids. The behavior of adolescents in southern Brazil may be reflecting cultural habits in a more urbanized city, resulting in adolescents being exposed to a higher prevalence of obesity and a sedentary lifestyle and ultra-processed foods [43].

We found that ethnicity was a predictor of change in insulin sensitivity, in which self-declared black adolescents were more likely to have IR. However, for girls aged over 15 years, this analysis did not remain significant. In this perspective, ethnicity has been associated with IR in adults and adolescents [2, 44, 45]. Indeed, preventing or reducing cardio-metabolic risk in African American girls has been recognized as an important public health objective [44]. Also, specifically in Brazil, socioeconomic inequality follows ethnic diversity, however with unclear repercussions on the health outcomes in Brazilian women at different stages of life [46, 47].

The limitations of this study include the possibility of reverse causality, because of its cross-sectional design. Furthermore, this study was conducted retrospectively using data collected for a larger research project. The strengths and relevant points of this study include new data on the prevalence of IR and its possible predictors in female adolescents from two regions in the geographical extremities of Brazil. Another methodological strength was the use of a hierarchical model that included socioeconomic and behavioral factors, nutritional status and association with time since menarche.

## Conclusions

In conclusion, data from this study indicate that IR is more prevalent in the peri-menarche, especially in younger and black adolescents, compared to white and post-menarche adolescents. Ow/Ob and high WC were associated with the occurrence of IR independently of age and ethnicity variables. Further research with a longitudinal design is warranted in order to confirm and extend the findings from this study. There is still a need for further clarification on causal associations between environmental and lifestyle factors and IR in adolescent girls from regions with diverse ethnic and socio-cultural characteristics.

## Supplementary Information

**Additional file 1: Table S1.** Prevalence ratios of insulin resistance according to cities and time since menarche in girls aged 12 to 17 years. **Table S2. **Prevalence ratio of insulin resistance, stratified by HOMA-IR and insulin in Porto Velho. **Table S3.** Prevalence ratio of insulin resistance, according to HOMA-IR and insulin in Porto Alegre. **Table S4.** Characterization of dietary variables according to insulin resistance, in Porto Velho and Porto Alegre, 2013-2014 (n=889).

**Additional file 2: Table S5.** Distribution of insulin resistance and insulin according to time of menarche, in the cities of Porto Velho-RO (n=382) and Porto Alegre-RS (n=507).

## Data Availability

Most of data generated or analyzed during this study are included within the article and its supplementary information file. Any additional data are available from the corresponding author on reasonable request.

## Abbreviations

IR: Insulin resistance
PVh: Porto Velho
PoA: Porto Alegre
HOMA-IR: Homeostasis model assessment for insulin resistance
DM: Diabetes mellitus
ERICA: Study of cardiovascular risks in adolescents
BMI: Body mass index
WC: Waist circumference
PR: Prevalence ratio
Ow/Ob: Overweight/obesity
