# Supplementary material for: Insulin resistance and associated factors in female adolescents from two capital cities in the north and south of Brazil
Source: Diabetol Metab Syndr. 2021 Oct 19;13:113. doi: 10.1186/s13098-021-00730-8 (PMC8527714; doi:10.1186/s13098-021-00730-8)
Supplement: Supplementary file 1 — Additional file 1: Table S1. Prevalence ratios of insulin resistance according to cities and time since menarche in girls aged 12 to 17 years. Table S2. Prevalence ratio of insulin resistance, stratified by HOMA-IR and insulin in Porto Velho. Table S3. Prevalence ratio of insulin resistance, according to HOMA-IR and insulin in Porto Alegre. Table S4. Characterization of dietary variables according to insulin resistance, in Porto Velho and Porto Alegre, 2013-2014 (n=889). [file 13098_2021_730_MOESM1_ESM.docx]

**Additional file 1: Table S1.** Prevalence ratios of insulin resistance according to cities and time since menarche in girls aged 12 to 17 years.

|  |  | **HOMA-IR >3.16** |  |  | **Insulin** ≥15mU/mL |  |
| --- | --- | --- | --- | --- | --- | --- |
|  | **PR** | **95%CI** | **P value** | **PR** | **95%CI** | **P value** |
| **City** |  |  |  |  |  |  |
| Porto Velho | 1 |  |  | 1 |  |  |
| Porto Alegre | 1.3 | (0.75-2.36) | 0.305 | 1.23 | (0.69-2.19) | 0.470 |
| **Time of menarche** |  |  |  |  |  |  |
| Less than 2 years since menarche  (peri-menarche) | 1 |  |  | 1 |  |  |
| More than 2 years since menarche  (post-menarche). | 0.53 | (0.35-0.78) | **0.002** | 0.49 | (0.33-0.74) | **0.001** |

Multivariate Poisson regression model; 95%CI.

**Additional file 1: Table S2.** Prevalence ratio of insulin resistance, stratified by HOMA-IR and insulin in Porto Velho.

|  |  | **Porto Velho (382)** | | | |
| --- | --- | --- | --- | --- | --- |
|  |  | **HOMA-IR**  **(61)** | | **Insulin**  **(58)** | |
|  |  | **Adjusted** | **Adjusted P value** | **Adjusted** | **Adjusted P value** |
| ***Level 1*** | **Age** |  |  |  |  |
|  | 12-13 years | 1 |  | 1 |  |
|  | 14 years | 0.65  (0.30-1.41) | 0.263 | 0.84  (0.32-2.21) | 0.711 |
|  | 15-17 years | 0.51  (0.28-0.92) | **0.028** | 0.63  (0.34-1.17) | 0.135 |
| ***Level 2*** | **Age at menarche**  ˂12 years | 2.17  (0.89-5.28) | 0.083 | 2.38  (0.88-6.46) | 0.082 |
|  | **Time since menarche**  ≥ 2 years | 0.38  (0.14-1.01) | **0.054** | 0.41  (0.15-1.11) | 0.077 |
|  | **Alcohol** |  |  |  |  |
|  | Yes | 1.79  (1.11-2.89) | **0.019** | 1.54  (0.88-2.70) | 0.116 |
|  | **Smoking** |  |  |  |  |
|  | Yes | 1.62  (0.75-3.53) | 0.201 | 2.02  (0.98-4.18) | 0.055 |
| ***Level 3***  ***Model 1*** | **Waist circumference** |  |  |  |  |
|  | Altered | 4.44  (2.55-7.72) | **˂0.001** | 4.29  (2.47-7.47) | **˂0.001** |
| **Level 3**  ***Model 2*** | **Nutritional status** |  |  |  |  |
|  | Overweight and obesity | 4.52  (2.12-9.63) | **0.001** | 5.16  (2.22-12.00) | **0.001** |

Multivariate Poisson regression model; P value ≤0.05 and 95%CI; IR = HOMA ≥3.16; Insulin ≥15mU/mL (I Diretriz de prevenção da aterosclerose na infância e adolescência, 2005); Group menarche ≥ 2 years: 2 years or more since menarche; Reference Category = 1 (Less than 2 years since menarche. Reference age at menarche more than 12 years; Alcohol: no = never experimented, and yes = already experimented; Smoking no = never experimented, and yes = already experimented. Altered waist circumference ˃90^th^ percentile or ≥80cm (IDF, 2005); nutritional status classification according to BMI-for-age z-score (WHO, 2007).

**Additional file 1: Table S3.** Prevalence ratio of insulin resistance, according to HOMA-IR and insulin in Porto Alegre.

|  |  | **Porto Alegre (507)** | | | |
| --- | --- | --- | --- | --- | --- |
|  |  | **HOMA-IR**  **(109)** | | **Insulin**  **(95)** | |
|  |  | **Adjusted** | **Adjusted P value** | **Adjusted** | **Adjusted P value** |
| ***Level 1*** | **Age** |  |  |  |  |
|  | 12-13 years | 1 |  | 1 |  |
|  | 14 years | 0.76  (0.42-1.35) | 0.338 | 0.82  (0.39-1.71) | 0.600 |
|  | 15-17 years | 0.62  (0.39-0.99) | **0.047** | 0.61  (0.35-1.06) | 0.082 |
| ***Level 2*** | **Ethnicity** |  |  |  |  |
|  | Black | 2.51  (1.32-4.78) | **0.007** | * | * |
|  | Others | 1.60  (0.98-2.62) | 0.058 | * | * |
|  | **Menarche Time**  ≥ 2 years | 0.65  (0.39-1.09) | 0.106 | 0.49  (0.32-0.77) | **0.003** |
|  | **Alcohol** |  |  |  |  |
|  | Yes | 0.77  (0.52-1.13) | 0.179 | * | * |
| ***Level 3***  ***Model 1*** | **Waist circumference** |  |  |  |  |
|  | Altered | 2.34  (1.61-3.38) | **˂0.001** | 2.55  (1.67-3.89) | **˂0.001** |
| **Level 3**  ***Model 2*** | **Nutritional status** |  |  |  |  |
|  | Overweight and obesity | 1.57  (1.10-2.23) | **0.014** | 1.94  (1.30-2.88) | **0.002** |

Multivariate Poisson regression model; P value ≤0.05 and 95%CI; IR = HOMA ≥3.16; Insulin ≥15mU/mL (I Diretriz de prevenção da aterosclerose na infância e adolescência, 2005); Ethnicity: white (reference), black and others (indigenous, mixed and yellow); Group Menarche ≥ 2 years: 2 years or more since menarche; Reference: category = 1 (Less than 2 years since menarche); Alcohol: no = never experimented, and yes = already experimented; Smoking no = never experimented, and yes = already experimented. Altered waist circumference ˃90^th^ percentile or ≥80cm (IDF, 2005); nutritional status classification according to BMI-for-age z-score (WHO, 2007); (*) p˃0.20.

**Additional file 1: Table S4.** Characterization of dietary variables according to insulin resistance, in Porto Velho and Porto Alegre, 2013-2014 (n=889).

|  | **HOMA-IR** | | **P value** | **Insulin** | | **P value** |
| --- | --- | --- | --- | --- | --- | --- |
| **Characteristics** | **Porto Velho (n=61)** | **Porto Alegre**  **(n=109)** |  | **Porto Velho**  **(n=58)** | **Porto Alegre**  **(n=95)** |  |
| ***Diet*** | **Mean (Standard error)** | **Mean (Standard error))** |  | **Mean (Standard error)** | **Mean (Standard error)** |  |
| **Energy (Kcal)** | 2456  (261) | 1841  (80) | **0.030** | 2618  (219) | 1786  (87) | **0.001** |
| **Omega-6: Omega-3 ratio** | 8.23  (0.30) | 8.37  (0.42) | 0.809 | 8.08  (0.30) | 7.95  (0.21) | 0.727 |
| **Trans-fat (g)** | 1.03  (0.13) | 1.20  (0.12) | 0.349 | 1.01  (0.12) | 1.26  (0.14) | 0.189 |

Student *t* test, mean and standard error; IR: HOMA-IR ≥3.16 and insulin ≥15mU/mL (I Diretriz de prevenção da aterosclerose na infância e adolescência, 2005); Nutrients expressed as daily intake.
