# Supplementary material for: Insulin resistance and associated factors in female adolescents from two capital cities in the north and south of Brazil
Source: Diabetol Metab Syndr. 2021 Oct 19;13:113. doi: 10.1186/s13098-021-00730-8 (PMC8527714; doi:10.1186/s13098-021-00730-8)
Supplement: Supplementary file 2 — Additional file 2: Table S5. Distribution of insulin resistance and insulin according to time of menarche, in the cities of Porto Velho-RO (n=382) and Porto Alegre-RS (n=507). [file 13098_2021_730_MOESM2_ESM.docx]

**Additional file 2: Table S5.** Distribution of insulin resistance and insulin according to time of menarche, in the cities of Porto Velho-RO (n=382) and Porto Alegre-RS (n=507).

| **Cities** | **HOMA-IR** | | | |  | **Insulin** | | | |  |
| --- | --- | --- | --- | --- | --- | --- | --- | --- | --- | --- |
|  | **<3.16** | **≥3.16** | **<3.16** | **≥3.16** | **P value** | **<15** | **≥15** | **<15** | **≥15** | **P value** |
|  | **Peri-menarche group** | | **Post-menarche group** | |  | **Peri-menarche group** | | **Post-menarche group** | |  |
| **PVh** | 71.27  (48.83-86.58) | 28.73  (13.42-51.17) | 86.75  (80.29-91.33) | 13.25  (8.67-19.71) | **0.031** | 75.7  (56.5-88.2) | 24.3  (11.8-43.5) | 86.33  (79.11-91.32) | 13.67  (8.68-20.89) | 0.064 |
| **PoA** | 64.28  (53.47-73.81) | 35.72  (26.19-46.53 | 80.85  (74.11-86.16) | 19.15  (13.84-25.89) | **0.005** | 66.73  (54.82-76.82) | 33.27  (23.18-45.18) | 84.44  (79.53-88.34) | 15.56  (11.66-20.47) | **0.002** |
| **Pvh/PoA** | 66.49  (56.82-79.95) | 33.51  (25.05-43.18) | 82.32  (77.23-86.47) | 17.68  (23.53-22.77) | **0.001** | 69.56  (59.84-77.79) | 38.44  (22.21-40.16) | 84.91  (81.02-88.11) | 15.09  (11.89-18.98) | **0.005** |

Chi-square; 95%CI; Group A = less than 2 years since menarche; Group B = more than 2 years since menarche.
